# Maternal UPD of chromosome 7 in a patient with Silver‐Russell syndrome and Pendred syndrome

**DOI:** 10.1002/jcla.23407

**Published:** 2020-07-14

**Authors:** Chuan Zhang, Shengju Hao, Qinghua Zhang, Furong Liu, Bingbo Zhou, Feng Xuan, Wang Xing, Xue Chen, Yan Wang, Panpan Ma, Zongfu Cao, Xu Ma

**Affiliations:** ^1^ Graduate School of Peking Union Medical College Beijing China; ^2^ National Research Institute for Family Planning National Human Genetic Resources Center Beijing China; ^3^ Gansu Province Medical Genetics Center Gansu Province Maternal and Child Health Care Hospital Lanzhou China

**Keywords:** growth retardation, PDS, SLC26A4, UPD7

## Abstract

**Background:**

Silver‐Russell syndrome (SRS) is a heterogeneous imprinting disorder featuring severe intrauterine and postnatal growth retardation and dysmorphic features. Pendred syndrome (PDS) is an autosomal recessive disorder caused by mutations in the *SLC26A4* gene characterized by sensorineural hearing loss.

**Methods:**

Karyotyping analysis was performed to investigate any chromosomal abnormalities. Whole‐genome copy number variation and loss of heterozygosity were analyzed using an Affymetrix CytoScan 750 K Microarray. Variant screening was performed by targeted next‐generation sequencing on all known deafness‐causing genes.

**Results:**

The proband was a patient with SRS caused by maternal uniparental disomy 7. The PDS of the proband was caused by homozygous variant c.919‐2A > G of *SLC26A4*; both mutated alleles were inherited from his mother.

**Conclusion:**

This is the first report of uniparental disomy 7 leading to SRS and Pendred syndrome. Patients with intrauterine growth retardation or those born small for gestational age and exhibiting postnatal growth failure should undergo molecular testing to reach a clinical diagnosis.

## 1 INTRODUCTION

Silver‐Russell syndrome (SRS, OMIM180860) is a clinically heterogeneous condition characterized by severe intrauterine growth retardation, poor postnatal growth, early feeding difficulties, craniofacial features such as a triangular face, and a broad forehead, body asymmetry, and a variety of minor malformations. The phenotypic expression changes during childhood and adolescence, with the facial features and asymmetry usually becoming more subtle with age. The incidence of SRS ranges from 1:30 000 to 1:100 000.^1^ Hypomethylation of the imprinted H19/IGF2 locus at 11p15 and maternal uniparental disomy (UPD) 7 are two major epigenetic etiologies in SRS.^2^, ^3^ Over 50% of SRS cases are caused by loss of paternal allele methylation (LOM) of imprinting H19/IGF2 center; variants in CDKN1C and IGF2, copy number variants, or mosaic segmental UPD of 11p15.5 are also associated with SRS; ^4^ and about 10% of SRS cases are due to maternal UPD7.^5^

Pendred syndrome (PDS), the most common type of syndromic deafness, is an autosomal recessive disorder associated with developmental abnormalities of the cochlea, sensorineural hearing loss, and diffuse thyroid enlargement (goiter).^6^ PDS can be caused by homozygous or compound heterozygous mutations in the SLC26A4 (OMIM 605646) gene on chromosome 7q22.3. PDS may also rarely be caused by digenic inheritance of a heterozygous mutation in the SLC26A4 gene and a heterozygous mutation in the FOXI1 gene (OMIM, 601093).^7^ Mutations in the SLC26A4 gene can also cause autosomal recessive deafness‐4 (DFNB4; 600791) with enlarged vestibular aqueduct.^8^

In 2018, Cirello^9^ reported a case exhibiting both SRS and PDS. The SRS was caused by segmental maternal UPD7q, and the PDS was caused by homozygous deletion of exons 17‐20 of SLC26A4, located at 7q. Here, we study the genetic etiology of a 4‐year‐old boy with severe developmental delay and hearing loss. By molecular diagnosis, the boy’s condition was shown to be caused by maternal UPD7. The proband was identified as having a homozygous variant c.919‐2A > G of SLC26A4, while only his mother carried this variant in heterozygous form. To our knowledge, this is the first case of SRS caused by maternal UPD7 accompanied by deafness caused by a homozygous variant of *SLC26A4*.

## 2 METHODS

### 2.1 Sample

**Figure 1:**
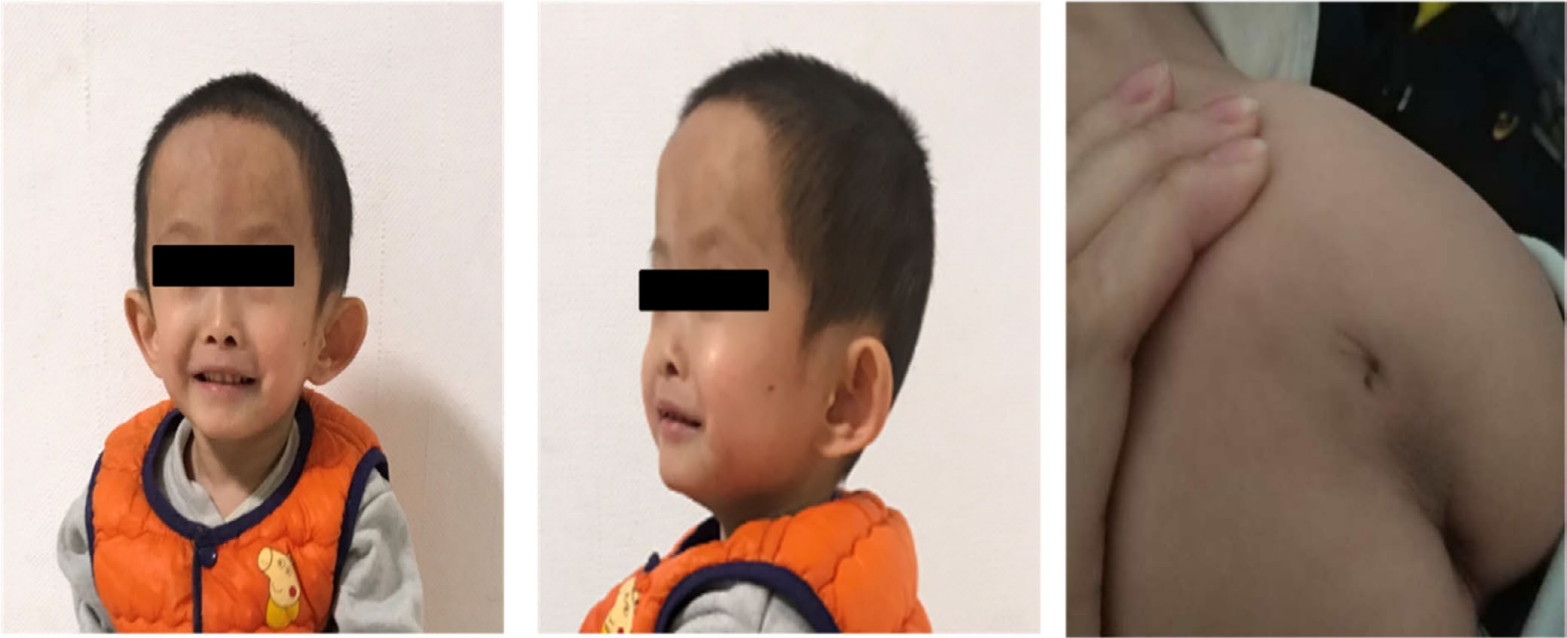
Clinical characterization of the case:high forehead, thin lips, low‐set posteriorly rotated ears, mild frontal bossing, and spina bifida

The proband was a 4‐year‐old boy born at 36 weeks by vaginal delivery. He was the first child of healthy nonconsanguineous parents of Chinese Han ethnicity. The birthweight was 1420 g, height was 36 cm, and head circumference was 24 cm. He was transferred to the neonatal intensive care unit because of asphyxia. At 73 days of age, his mental reaction was acceptable, and there were no signs of irritability or convulsions, no apnea, oxygen saturation >80%, and normal blood pressure; the patient was thus discharged from hospital. Hearing loss was first noted during hospitalization by binaural hearing screening. At 4 years of age, the proband visited our hospital for genetic counseling because of severe developmental delay, with height 75 cm and weight 6500 g. Craniofacial dysmorphic features included high forehead, thin lips, low‐set posteriorly rotated ears, and mild frontal bossing (Figure 1), along with other features including spina bifida (Figure 1). This study was performed in accordance with the tenets of the Declaration of Helsinki. This study was also approved by the Ethics Committee of Gansu Provincial Maternal and Child Health Care Hospital. Written informed consent for the publication of this report was obtained from the proband’s parents.

### 2.2 Cytogenetic analysis

Peripheral venous blood samples were obtained from the proband and his parent. Routine karyotyping was performed on GTG‐banded metaphases from cultures of PHA‐stimulated peripheral blood lymphocytes, in accordance with standard procedures. Karyotypes were classified in line with the International System for Human Cytogenetic Nomenclature 2016.^10^ At least 20 metaphases per sample were analyzed whenever possible.

### 2.3 Sample collection and genomic DNA preparation

Blood samples of 2**–**3 mL were collected from the proband and his parents. Genomic DNA was extracted using Tiangen Biotech DNA extraction kit and quantified spectrophotometrically using a NanoDrop 2000 spectrophotometer (Thermo Scientific). The DNA concentration was no <50 ng/µL, OD_260/280_ was about 1.9, and OD_260/230_ was about 2.0.

### 2.4 Target gene capturing and sequencing and Sanger sequencing

We used targeted gene capturing and sequencing (MyGenostics GenCap Enrichment Technologies) to screen the mutations in 121 genes that had been reported to cause hereditary hearing loss. PCR primers for c.919‐2A > G of *SLC26A4* (NM_000441.1) were designed by Primer 3.0 (http://bioinfo.ut.ee/primer3‐0.4.0/); the forward primer was 5′‐AAGTTCAGCATTATTTGGTTGACA‐3′ and the reverse primer was 5′‐TGGTTGTTTCTTCCAGATCACA‐3′. PCR conditions for c.919‐2A > G of SLC26A4 were as follows: 95°C for 5 minutes, followed by 35 cycles of 94°C for 30 seconds, 56°C for 45 seconds, and 72°C for 45 seconds. The DNA sequencing was performed on an ABI 3500DX Genetic Analyzer (Applied Biosystems).

### 2.5 CytoScan 750 K Microarray assay

Whole‐genome CNV analysis was performed using Affymetrix CytoScan 750 K Microarray (Thermo Scientific), which contains 550 000 markers for detecting copy number variation and 200 000 high‐performing SNP probes, and can perform array‐CGH and SNP‐array at the same time. The workflow of the CytoScan^™^ assay was performed in accordance with the Affymetrix^®^ CytoScan^™^ Assay protocol.^11^

### 2.6 Microsatellite analysis

Microsatellite analysis was performed using five STR markers (D7S460, D7s821, D7s1818, D7S1804, and D7S2446) spanning the whole of chromosome 7.^12^ PCR amplicons were genotyped on capillary electrophoresis using the 3 ABI 3500DX Genetic Analyzer (Applied Biosystems). Data analysis was performed using GeneMapper4 software (Applied Biosystems) matching parental to proband transmission.

## 3 RESULTS

**Figure 2:**
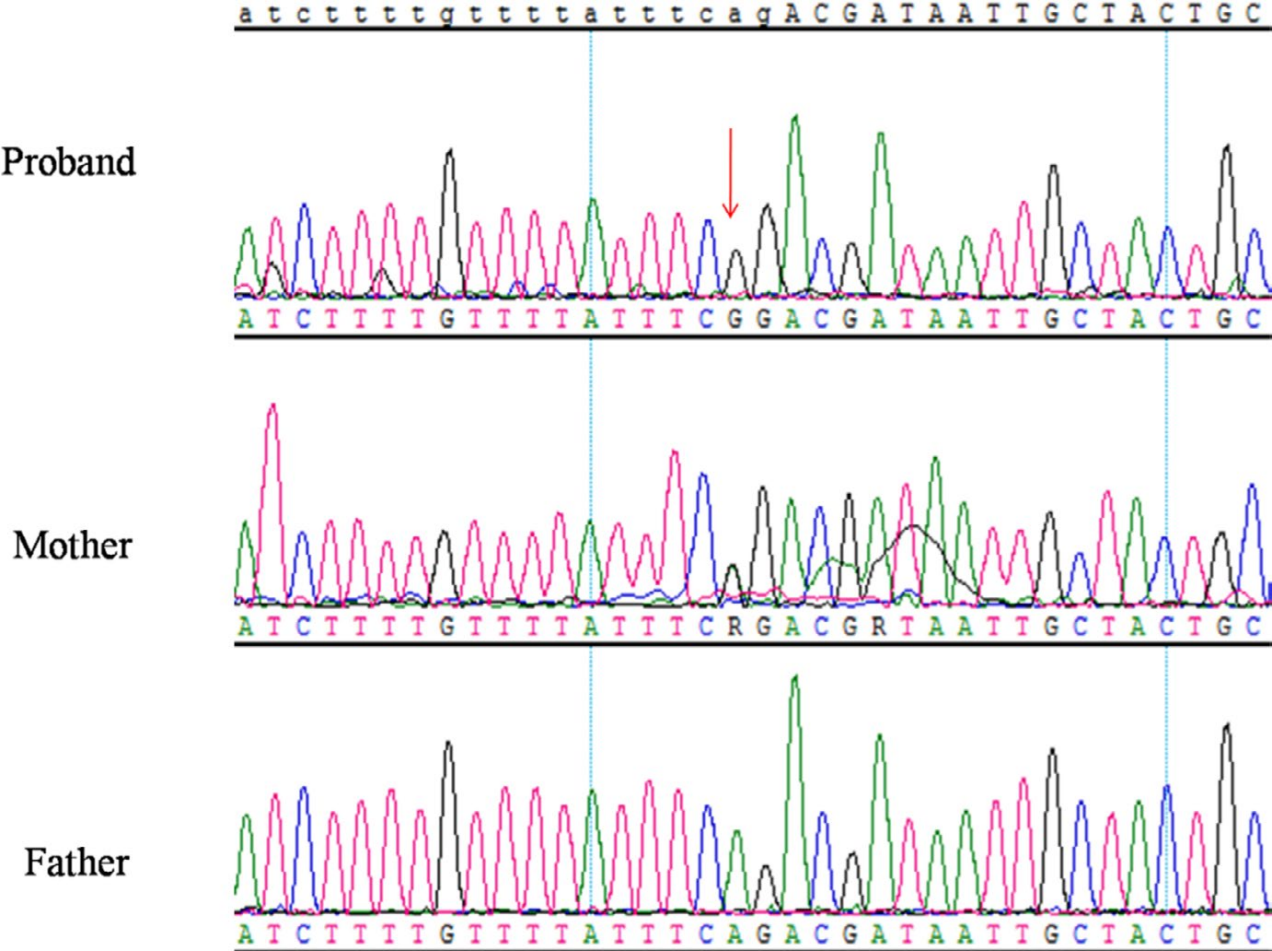
Results of Sanger sequencing: the proband detected homozygous variant c.919‐2A > G/c.919‐2A>G of SLC26A4, mother detected heterozygous variant c.919‐2A > G; father did not find heterozygous variant c.919‐2A > G

The karyotypes of the proband and his parents were normal. The variant c.919‐2A > G of SLC26A4 in homozygous form was identified through targeted gene capturing sequencing in the proband. The heterozygous variant c.919‐2A > G was identified in the proband’s mother but not in his father by Sanger sequencing (Figure 2).

**Figure 3:**
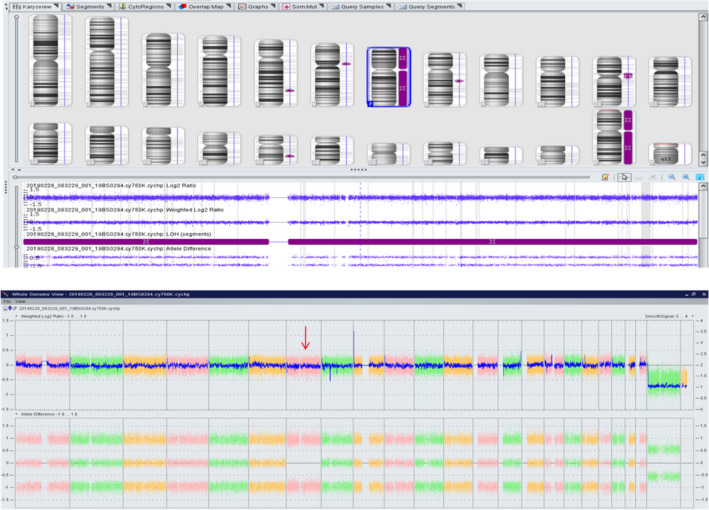
The molecular‐cytogenetic analyses of the proband by Affymetrix CytoScan 750 K Microarray

The results of molecular/cytogenetic analyses of the proband were as follows: arr[hg19] arr(1‐22)x2, (XY) x1; arr[hg19] 7p22.3p11.1 (50 943‐58 019 983) hmz; arr[hg19] 7q11.21q36.3 (62 569 501‐159 118 443) hmz (`ure 3), which suggested that the proband has UPD for chromosome 7 (Figure 3). Maternal UPD7 was indicated, in accordance with the results of microsatellite analysis (Table 1).

**Table 1:** The result of microsatellite analysis

	D7S460	D7s821	D7s1818	D7S1804	D7S2446
Proband	170; 170	257; 257	182*; 182*	276*; 276*	191*; 191*
Father	170; 174	257; 265	186; 190	256; 272	187; 200
Mother	170; 182	257; 261	182*; 190	276*; 276*	191*; 191*

* Only found in proband and mother.

## 4 DISCUSSION

We identified the etiology of a boy suffering from severe developmental delay and hearing loss. His weight (1420 g) and height (36 cm) were less than −2 SD for his gestational age at birth. At 4 years of age, he was still presenting with severe growth retardation, with height of 75 cm and weight of 6500 g. He had certain craniofacial dysmorphic features, such as high forehead, thin lips, low‐set posteriorly rotated ears, and mild frontal bossing, as well as other features including spina bifida. In accordance with the Netchine‐Harbison clinical scoring system,^1^ and combining the clinical features and molecular testing results, we diagnosed our proband with SRS, which was caused by maternal UPD7. The cause of the hearing loss of the proband is the homozygous variant c.919‐2A > G of *SLC26A4*, which is a pathogenic variant.^13^ Because the proband had received both copies of chromosome 7 from his mother, he presented this variant in homozygous form.

The incidence of SRS was reported to be about 1:30 000 to 1:100 000;^1^ however, only about 10% of SRS cases are due to maternal UPD7.^5^ PDS is the most common syndromal form of deafness and can be caused by homozygous or compound heterozygous mutation in *SLC26A4*, which is located on chromosome 7q22.3. Occasionally, recessive disorders may occur as a consequence of duplication or UPD of a region including a heterozygous pathogenic variant.^9^

The first international consensus statement on the diagnosis and management of SRS was published in 2016.^1^ SRS has variable characteristics, although it is easy to recognize, with typical symptoms such as severe postnatal growth delay and postnatal macrocephaly.^14^, ^15^ However, the diagnosis of SRS can be difficult as the condition varies widely in severity among affected individuals and many of its features are nonspecific.^16^, ^17^, ^18^ A positive molecular test can provide useful confirmation of the clinical diagnosis of SRS and can guide appropriate management of this condition. Over 30 different pathogenic CNVs have been reported in patients with suspected SRS.^19^, ^20^, ^21^, ^22^ SNP‐array^10^ and CNV‐seq^23^, ^24^ are the main methods for performing whole‐genome CNV analysis; however, at present, CNV‐seq cannot detect LOH variants. Because SRS can be caused by UPD of 11p15.5 and maternal UPD7, SNP‐array might be a better choice for patients with suspected SRS.

As far as we know, only two cases have been reported in the PubMed database (https://www.ncbi.nlm.nih.gov/pubmed/). Bigoni^25^ reported a patient affected by SRS who also presented with sensorineural hearing loss, but the genetic basis of the hearing loss in that patient was not explained. Additionally, Cirello et al.^9^ reported an SRS case with PDS caused by segmental maternal UPD7q and homozygous deletion of SLC26A4 exons 17‐20. Similar to this latter case, the PDS of our patient with SRS was caused by the maternal homozygous variant of SLC26A4, located at chromosome 7q22.3; the maternal UPD7 caused the homozygous state of the maternal variant. However, in contrast to the case reported by Cirello et al., our SRS was caused by maternal UPD of the whole of chromosome 7. The SRS phenotype of maternal UPD7 was thought to be caused by altered expression of an imprinted growth regulatory gene.^1^ Candidate SRS regions have been suggested through the identification of patients with segmental maternal UPD7, and the primary candidate genes on chromosome 7 are currently GRB10 (7p12.1) and MEST (7q32).^1^ In addition to the typical clinical features of SRS, we also found spina bifida in our patient; however, we did not find any gene on chromosome 7 reported to be associated with a predisposition for spina bifida, so we suspected that this might have been a coincidence.

In conclusion, this is the first report of UPD7 leading to SRS and PDS. Via molecular testing, we identified the etiology of the proband. SRS is rare and presents with prenatal and postnatal growth retardation. Molecular tests are recommended for patients with intrauterine growth retardation or those born small for gestational age and exhibiting postnatal growth failure. If a homozygous mutation is identified in a proband but only one parent is a carrier, screening for UPD by SNP‐array and microsatellite analysis is advisable.
